# Advances in the Synthesis and Analysis of Biologically Active Phosphometabolites

**DOI:** 10.3390/ijms24043150

**Published:** 2023-02-05

**Authors:** Roland Wohlgemuth

**Affiliations:** 1 MITR, Institute of Applied Radiation Chemistry, Faculty of Chemistry, Lodz University of Technology, Zeromskiego Street 116, 90-924 Lodz, Poland; roland.wohlgemuth.1@p.lodz.pl or roland.wohlgemuth@sk-biotechnologie.ch; 2 Swiss Coordination Committee Biotechnology (SKB), 8021 Zurich, Switzerland

**Keywords:** phosphorus cycle, biologically active metabolites, phosphometabolites, analytical advances, phosphometabolome

## Abstract

Phosphorus-containing metabolites cover a large molecular diversity and represent an important domain of small molecules which are highly relevant for life and represent essential interfaces between biology and chemistry, between the biological and abiotic world. The large but not unlimited amount of phosphate minerals on our planet is a key resource for living organisms on our planet, while the accumulation of phosphorus-containing waste is associated with negative effects on ecosystems. Therefore, resource-efficient and circular processes receive increasing attention from different perspectives, from local and regional levels to national and global levels. The molecular and sustainability aspects of a global phosphorus cycle have become of much interest for addressing the phosphorus biochemical flow as a high-risk planetary boundary. Knowledge of balancing the natural phosphorus cycle and the further elucidation of metabolic pathways involving phosphorus is crucial. This requires not only the development of effective new methods for practical discovery, identification, and high-information content analysis, but also for practical synthesis of phosphorus-containing metabolites, for example as standards, as substrates or products of enzymatic reactions, or for discovering novel biological functions. The purpose of this article is to review the advances which have been achieved in the synthesis and analysis of phosphorus-containing metabolites which are biologically active.

## 1. Introduction

Small molecular weight compounds containing phosphorus, which does not occur as a free element in nature, represent essential components of biological cells acting as substrates of enzymes or the products of enzymatic reactions, regulatory or signalling molecules, or building blocks for the biosynthesis of biopolymers, natural products, and cofactors [1]. The importance of phosphorus-containing metabolites (phosphometabolites) which are biologically active is related to the versatile phosphorus chemistry and derives from the key requirements for the element phosphorus as part of cellular components of living organisms, from microorganisms, plants, and animals to humans.

A detailed molecular understanding of the phosphometabolites and their biological activities, transformations, and storage is of much fundamental interest. This is also relevant in the broader context of the biochemical flow of phosphorus on our planet, which has been listed as one of the planetary boundaries to be concerned about, as the flow of phosphorus into the oceans is already approaching the proposed planetary boundary of 11 million tons per year [2]. After phosphorus was- discovered as an element in the 17th century, the evolution of human use of phosphorus has involved both positive and negative consequences, due to phosphorus being essential for sustaining all life and food production [3]. The privileged role of the element phosphorus in the living world is very clearly evident from the wide occurrence of phosphoric acid derivatives everywhere in biological organisms [4], for example in many important biological cell components, such as sugar phosphates, phosphate esters, and thioesters, phosphoric acid anhydrides, phospholipids, teichoic acids, cofactors, phosphoramidates, and nucleic acids. The flexibility and great functional variety of phosphates are essential for life and have been described by Lord Todd in his essay “Where There’s Life There’s Phosphorus” [4]. The question has been raised of how phosphate could have assumed these key roles and whether ancient metabolic networks without phosphate could have played a role in the origin of life on the primordial earth, taking into account the poor geochemical accessibility of phosphate [5]. The multiple functions of negatively charged phosphate esters and anhydrides, which are stable but still reactive under enzymatic catalysis, and retained by biological membranes, have been of great fundamental interest [6,7,8] and also provide inspiration for its utilization in organic chemistry [9].

The growing anthropogenic activities are affecting the global phosphorus cycle by increased mining of mineral phosphorus resources, inefficient utilization of phosphorus resources, and the influx of phosphorus waste into aqueous systems. Therefore, improvements in the biochemical flows and the resource efficiency of phosphorus are necessary for a sustainable phosphorus cycle. Among the complex challenges for which solutions need to be developed and implemented in moving from linear to circular processes are phosphorus recovery, recycling, and transformations which utilize renewable phosphate resources for sustainable manufacturing [10]. The rich phosphorus chemistry and the methods used by nature can provide inspiration for novel reaction discovery and the transition towards sustainable phosphorus chemistry, with the opportunity to avoid toxic intermediates and reduce energy input and waste [9,11]. This requires not only a macroscopic analysis of the flow of the element phosphorus, but also a molecular path analysis of organic and inorganic compounds, as well as the development of novel synthetic tools and methodologies.

A balanced biochemical flow of phosphorus is not only relevant at the planetary level, but also at the regional, national, and local levels. On the level of biological organisms, their health and risk of disease are also affected by phosphorus metabolism, and a major part of metabolites which are highly important for cellular metabolism, are stable kinetically and can be activated thermodynamically, are phosphorus-containing metabolites [12]. Avoiding that the phosphorus uptake by food and beverages with high phosphorus content exceeds very much the metabolic needs and secretion of phosphorus and maintaining phosphate metabolism in balance is also important for human health and the prevention of organic dysfunction and accelerated aging [13].

Phosphometabolites represent a significant fraction of the total number of metabolites, the metabolome, of microorganisms, plants, animals, and humans. The Human Metabolome Database HMDB 5.0 lists more than 30,000 phosphometabolites among its more than 220,000 endogenous metabolites [14]. While the discovery of many biologically active phosphometabolites as well as their analysis and synthesis represent important biochemistry milestones and there is still more work ahead, it is worthwhile to have a look at the current status of the analysis and synthesis of biologically active phosphorus-containing metabolites, for which a range of new tools and methodologies have been developed in various classes.

## 2. Structures of Biologically Active Phosphometabolites

**Figure 1:**
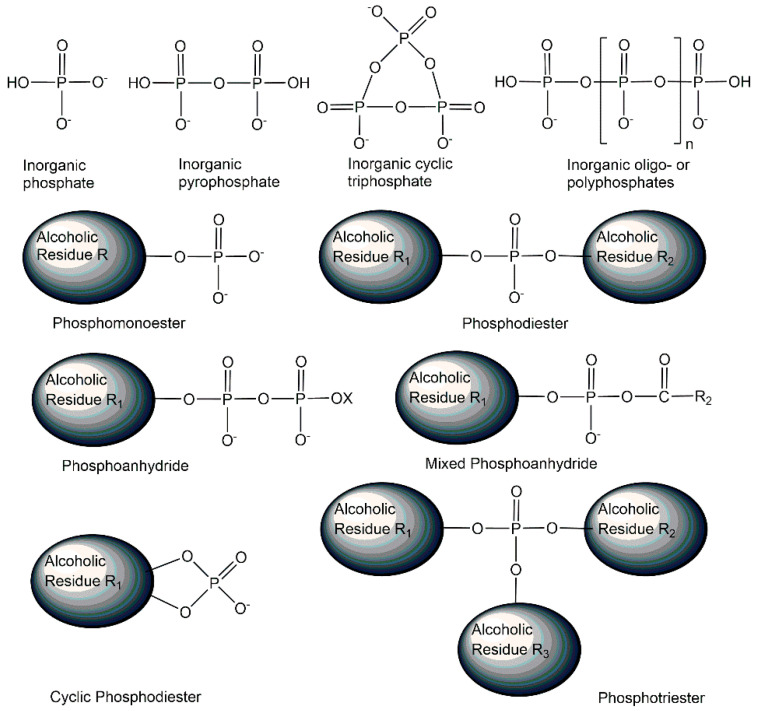
Selected phosphometabolite structures containing pentavalent phosphorus in oxidation state +5 and covalent phosphorus-oxygen bonds.

The element phosphorus is very versatile in undergoing chemical bonds with many elements, from hydrogen, boron, carbon, nitrogen, oxygen, and fluorine to sulfur, chlorine, bromine, iodine, or phosphorus itself, and can have oxidations states between +5 and −3 as well as coordination numbers between 1 and 9 [15]. At the earth’s surface, in minerals, and in the current metabolic pathways existing in the biosphere, phosphorus with the oxidation state +5 is largely predominant [16,17,18], but lower oxidations states and trivalent phosphorus are also of much interest in modern main group chemistry and as biologically active metabolites in organisms, for example in the exploration of the phosphonate biochemistry [19]. Nature’s focus on pentavalent phosphorus with an essentially constant oxidation state +5 and covalent phosphorus-oxygen bonds enables a wide structural diversity of phosphorus-containing metabolites (see Figure 1), from inorganic phosphates, pyro-, meta- and polyphosphates to phosphoanhydrides, phosphomonoesters, phosphodiesters, and phosphotriesters [1].

**Figure 2:**
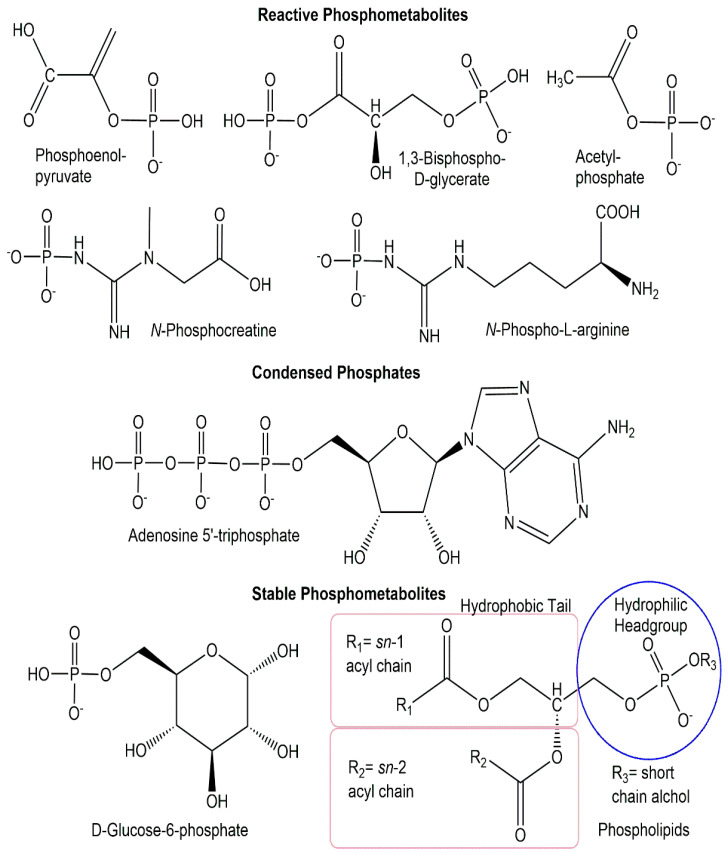
Selected phosphometabolite structures from the three energy classes of reactive phosphorus compounds, condensed phosphates, and stable phosphorus compounds.

From an energetic perspective, the diversity of phosphometabolites can also be divided into three energy classes (see Figure 2): reactive phosphorus compounds, condensed phosphates, and stable phosphorus compounds [20].

**Figure 3:**
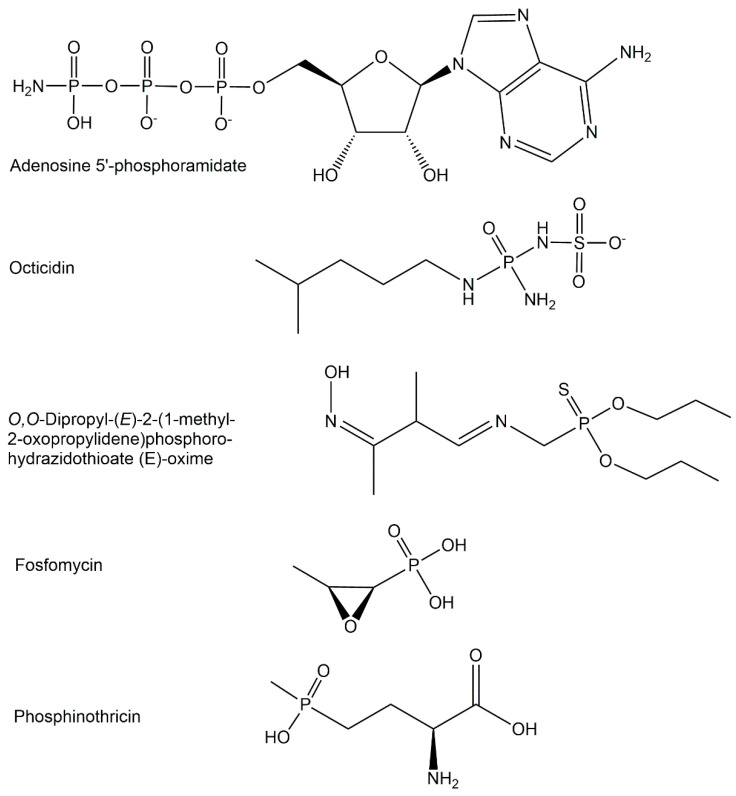
Selected phosphometabolite structures containing *P-N-*bonds, *P-S-*bonds, and *P-C-*bonds.

The majority of biologically active phosphometabolites identified so far are phosphate derivatives containing phosphorus-oxygen bonds, as discussed previously. Important phosphometabolites can, however, also be found among phosphate derivatives containing a *P-N-*bond, a *P-S-*bond, or a *P-C-*bonds instead of a *P-O-*bond, or a combination thereof. The replacement of one of the four phosphate oxygens by nitrogen, sulfur, or carbon (see Figure 3) can be found in the phosphometabolite classes of phosphoramidates, phosphorothioates, and phosphonates [21].

Phosphometabolite structures can also be put into the context of their biological activities in living cells. Intrinsic phosphometabolites of the corresponding cells play key roles as endogenous phosphometabolites in the proper functioning and regulation of central carbon, energy, and phosphorus metabolism. Phosphometabolites derived from the transport of extrinsic compounds to the corresponding cells and subsequent intracellular modification are different from cellular phosphometabolites and can act as important exogenous phosphometabolites in the treatment of diseases by pharmaceuticals.

### 2.1. Endogenous Phosphometabolites

**Figure 4:**
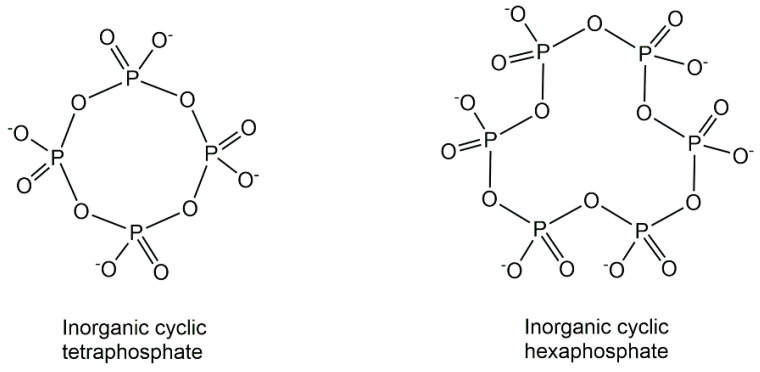
Natural inorganic cyclic tetra-and hexaphosphate structures.

The natural inorganic phosphometabolites, originating from the condensation of phosphates and consisting only of phosphorus and oxygen, show a fascinating richness of structures (see Figure 4) ranging from simple inorganic phosphate to various linear, branched (ultraphosphates) and cyclic (metaphosphates) structures of polyphosphates [22]. In addition to linear polyphosphates, substantial amounts of small cyclic polyphosphate structures have been discovered in phosphate granules of *Xanthobacter autotrophicus* by using non-destructive solid-state ^31^P NMR methods [23]. Based on the new synthetic access to defined branched short-chain ultraphosphates, which has enabled investigations of their properties and reactivity, surprising stabilities with half-lives up to days have been discovered [24].

**Figure 5:**
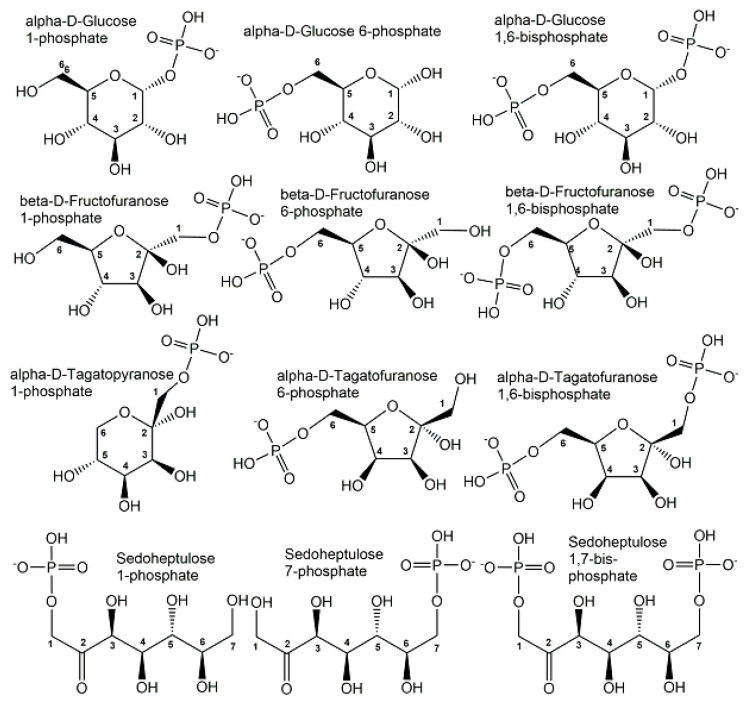
Selected endogenous mono- and bis-phosphorylated monosaccharide structures, with their phosphorylation sites designated by the corresponding carbon atom numbering.

The majority of organic phosphometabolites contain covalent bonds of phosphorus to oxygen or nitrogen. Phosphorylated derivatives of monosaccharides and polyols show great structural diversity and are key metabolic intermediates and regulators of carbohydrate metabolism. As the monophosphorylation of neutral sugars and polyols is already sufficient for keeping phosphometabolites inside cells, it is not surprising that the number of phosphate monoesters of monosaccharides and polyols carrying one phosphoryl group is much larger than the ones carrying two or more phosphoryl groups. Although the number of bis-phosphorylated monosaccharides is smaller, some important biological activities are connected with them, such as the bis-phosphorylated forms of D-glucose, D-fructose, D-tagatose, and D-sedoheptulose (see Figure 5).

**Figure 6:**
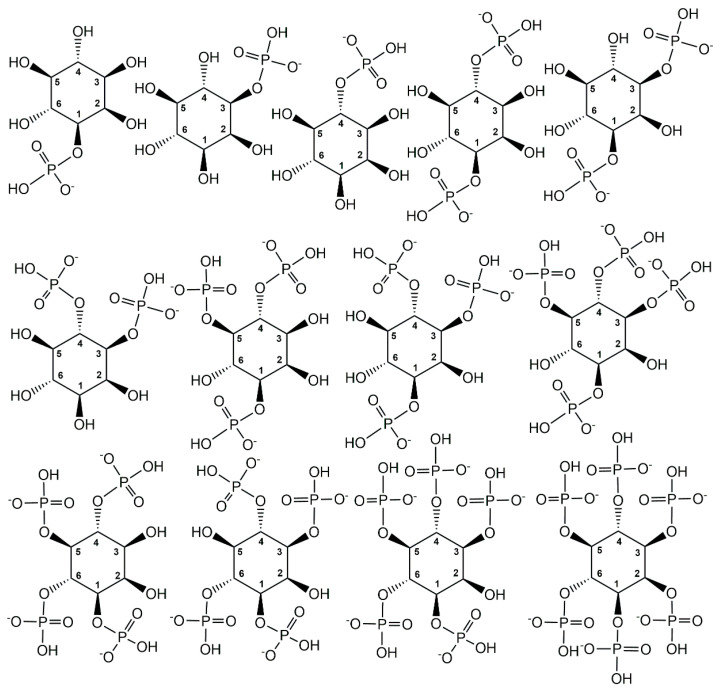
Structures of biologically active mono-, bis- tris-, tetra-, penta- and hexa-phosphorylated *myo*-inositols. Different *myo*-inositol phosphometabolites and their stereoisomers can be recognized by the carbon atom numbering of their phosphorylation sites.

The tris- and tetra-phosphorylated myo-inositols, which can occur with different substitution patterns according to their specific phosphorylation sites at three or four of the hydroxy groups, are important signalling compounds. It is also special that biologically active *myo*-inositol phosphometabolites cover the whole range from mono- to hexaphosphorylated *myo*-inositols (see Figure 6).

**Figure 7:**
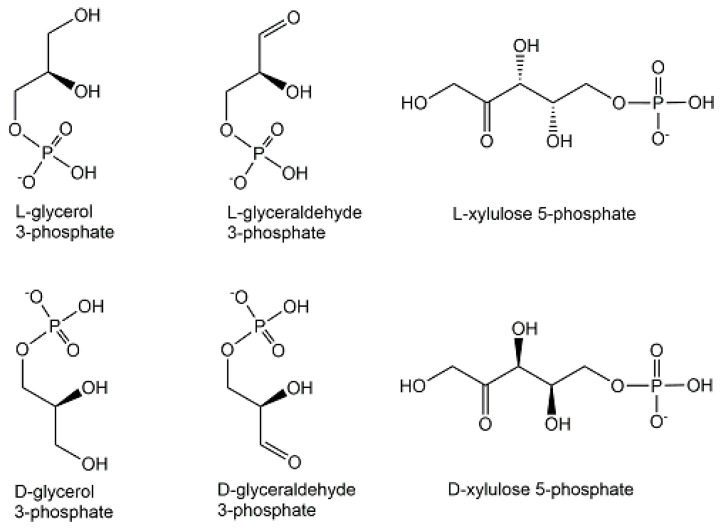
Enantiomers of terminally phosphorylated glycerol, glyceraldehyde, and xylulose.

An important feature of structural diversity comes from the chirality of non-symmetrical phosphometabolites, whether this originates from an already chiral non-phosphorylated precursor or is created by the desymmetrization of a symmetric non-phosphorylated precursor by phosphorylation. The molecular complexity and stereochemical diversity of phosphometabolites are increasing with the number of chiral centers and are of much interest for interactions with inherently chiral biomacromolecules in chemical biology [25]. Important examples of biologically active enantiocomplementary phosphate mono-esters carrying one phosphoryl group are the enantiomers of terminally phosphorylated glycerol, glyceraldehyde, and xylulose (see Figure 7). Stereocontrolled biosynthesis of chiral phosphometabolites in enantiocomplementary pathways is of much interest [26] and can be achieved by different enzymes from their corresponding non-phosphorylated chiral precursors, or by different enzymes from a common achiral precursor. Examples for the latter case are enzymatic reductions of dihydroxyacetone phosphate to the two enantiomeric forms of terminally phosphorylated glycerol (see Figure 7) catalyzed by enantiocomplementary dehydrogenases. The L-enantiomer of glycerol 3-phosphate, also named *sn*-glycerol-3-phosphate, or D-glycerol 1-phosphate, is formed by *sn*-glycerol 3-phosphate dehydrogenase-catalyzed reduction of dihydroxyacetone phosphate, while the D-enantiomer of glycerol 3-phosphate, also named as *sn*-glycerol-1-phosphate, or L-glycerol 1-phosphate, is formed by *sn*-glycerol 1-phosphate dehydrogenase-catalyzed reduction of dihydroxyacetone phosphate [27].

As monosaccharides and polyols are important carbon and energy sources for biological cells, their respective phosphometabolites are highly important in the central metabolism and regulation of healthy biological cells. These phosphate monoester structural features occur also in a variety of other well-known endogenous phosphometabolites, such as aminosugar phosphates, deoxysugar phosphates, phosphorylated sugar acids, phospholipids, and ribo- and deoxyribonucleoside monophosphates.

Phosphate monoesters are also involved in the RubisCO-catalyzed formation of 3-phospho-D-glycerate, a key reaction in the dominant biological carbon dioxide fixation on our planet converting >90% of inorganic carbon into biomass [28]. Constructing and optimizing novel synthetic biocatalytic systems is an exciting new area to overcome bottlenecks in biological carbon dioxide fixation, where phosphometabolites are involved either directly with phosphenolpyruvate in the synthetic rGPS cycle and the MCG pathway [29], or indirectly with the phosphorus-containing metabolites ATP, coenzyme A and NADP in the synthetic CETCH cycle [30].

**Figure 8:**
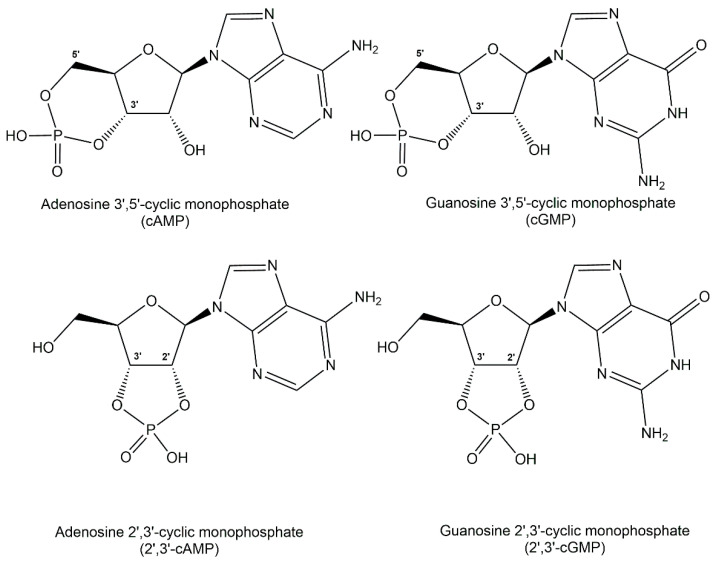
Structure of the nucleotide cyclic monophosphates AMP and cGMP as well as 2′,3′-cAMP and 2′,3′-cGMP.

Thermodynamic stability of phosphodiester structures is essential for many important biologically active phosphometabolites. The diacyl-sn-glycerol-3-phosphates esterified with polar head groups such as choline, ethanolamine, glycerol or L-serine in phospholipids are key for the integrity of cell membranes. Other phosphodiesters contain polar head groups such as choline and ethanolamine just esterified with sn-glycerol-3-phosphate. Phosphodiesters of polyols have been identified in extremophiles as osmolytes, also designated as extremolytes, such as di-*myo* inositol 1,1′-phosphate, diglycerol-phosphate, and glycerophospho-*myo*-inositol. The key functions as second messengers of the nucleotide 3′,5′-cyclic monophosphate signalling molecules are well established in archaea, bacteria, and eukarya, with the adenosine 3′,5′-cyclic monophosphate (cAMP) and the guanosine 3′,5′-cyclic monophosphate (cGMP), shown in Figure 8, acting as the most common second messengers [31]. The biological functions of the cytidine 3′,5′-cyclic monophosphate (cCMP) and the uridine 3′,5′-cyclic monophosphate (cUMP) as second messengers involved in bacterial immunity signalling against viruses have only recently been assigned [32]. The positional isomers adenosine 2′,3′-cyclic monophosphate (2′,3′-cAMP) and guanosine 2′,3′-cyclic monophosphate (2′,3′-cGMP) of the common cAMP and cGMP (see Figure 5) have recently been demonstrated to play key roles as signalling molecules in the immune response and cell death of plants mediated by the bifunctional plant TIR proteins which catalyze its formation [33,34]. Another important family of second messengers are the cyclic dinucleotides such as cyclo-diAMP, cyclo-diGMP, 3′,3′-cyclo-GAMP, or 2′,3′-cyclo-GAMP, which contain two phosphodiester moieties and have many signalling functions towards a variety of biological processes [35,36]. A common defense system against a wide range of phages has been demonstrated to involve cycloGAMP [37]. Cyclic dinucleotide analogues have attracted much therapeutic interest due to their biological activities, such as signalling molecules in immune response, especially analogues of the endogenous immune stimulant 2′,3′-cycloGAMP [38].

Energy-rich phosphoanhydride bonds are key to a range of central phosphometabolites, from simple pyro- or diphosphate to nucleoside diphosphates, nucleoside diphosphate sugars, prenyl diphosphates, 5-phospho-D-ribose-1-diphosphate, thiamine diphosphate, each containing one phosphoanhydride bond, to 2′-deoxynucleoside triphosphates and nucleoside triphosphates, among which ATP is the most prominent one [39], containing two phosphoanhydride bonds. Phosphoanhydride bonds are also present in the osmolyte cyclic 2,3-diphosphoglycerate, and in a number of signalling molecules, such as cyclic ADP-ribosides, or alarmone nucleotides such as ppGpp.

**Figure 9:**
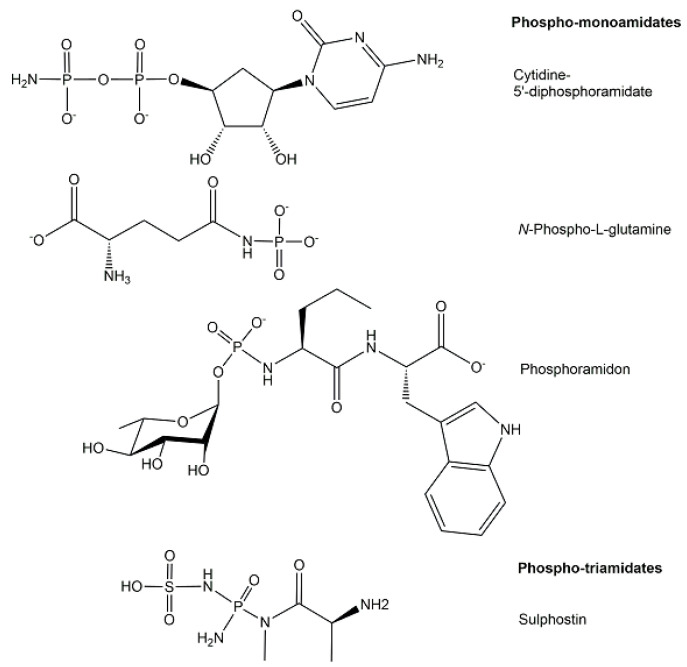
Selected structures of endogenous phosphoramidates from the classes of phosphomono-amidates and phospho-triamidates.

The structures of phosphoramidates, which are characterized by a single covalent bond between nitrogen and phosphorus with an oxidation state of +5 and coordination number 4, can be classified according to the type of amine bound to phosphorus (see Figure 9) into three different classes of *N*-phosphorylated compounds [21] where phosphorus is covalently bonded to the nitrogen (a) of a mono-substituted NH_2_-group, such as phosphagens or *N*-phosphorylated amino acids [40], (b) of a di-substituted NH_2_-group, and (c) of a free NH_2_-group, such as nucleoside 5′-phosphoramidates [41].

An increasing number of phosphoramidates have been discovered as biologically active phosphometabolites in microorganisms [21], such as cytidine diphosphoramidate as a metabolic intermediate in the biosynthesis of *Campylobacter jejuni* capsular polysaccharide [42]. Although the structure of the microbial metabolite phosphoramidon from *Streptomyces tanashiensis* was reported 50 years ago [43], its biosynthesis and powerful enzyme inhibitor properties towards various metalloendopeptidases, and endothelin-converting enzymes continue to be of interest [44]. The *P*-*N* bond can also be found in the structures of naturally occurring nucleotide antibiotics, for example in the phosmidosin from *Streptomyces durhameusis*, which has antifungal activity and an *O*-methylated phosphoramidate structure [40], and in the ribosomal peptide antibiotic microcin C7 from Escherichia coli, having the peptide at its C-terminus linked by a phosphoramidate linkage to adenosine monophosphate [45].

**Figure 10:**
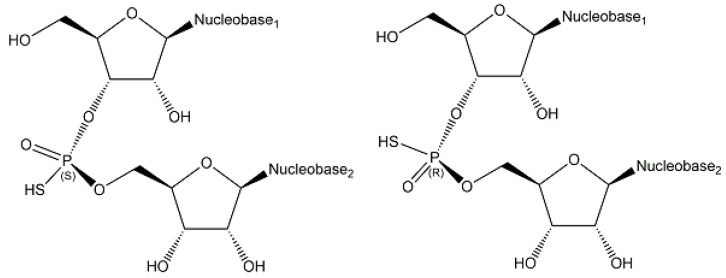
Selected phosphorothioate-linked dinucleotides.

Endogenous phosphorothioates have been rarely described [21], but *S*-phosphorylation has been demonstrated in the endogenous ENITNLDApCITR peptide from *E. coli* [46]. The naturally occurring sequence- and stereospecific introduction of sulfur into phosphate groups of the DNA backbone, which has been investigated by analyzing all 16 possible phosphorothioate-linked dinucleotides, has demonstrated that d(A_PS_A), d(C_PS_A), d(G_PS_A), d(G_PS_T), d(G_PS_G), and d(T_PS_A) (see schematic structures and stereochemistry in Figure 10) are phosphorothioate modifications of DNA, which stabilize against nuclease degradation and are widespread in bacteria [47,48]. So far, only the *R*_P_-stereoisomer, which results from stereospecifically replacing in a phosphate group a non-bridging oxygen by sulfur [47].

**Figure 11:**
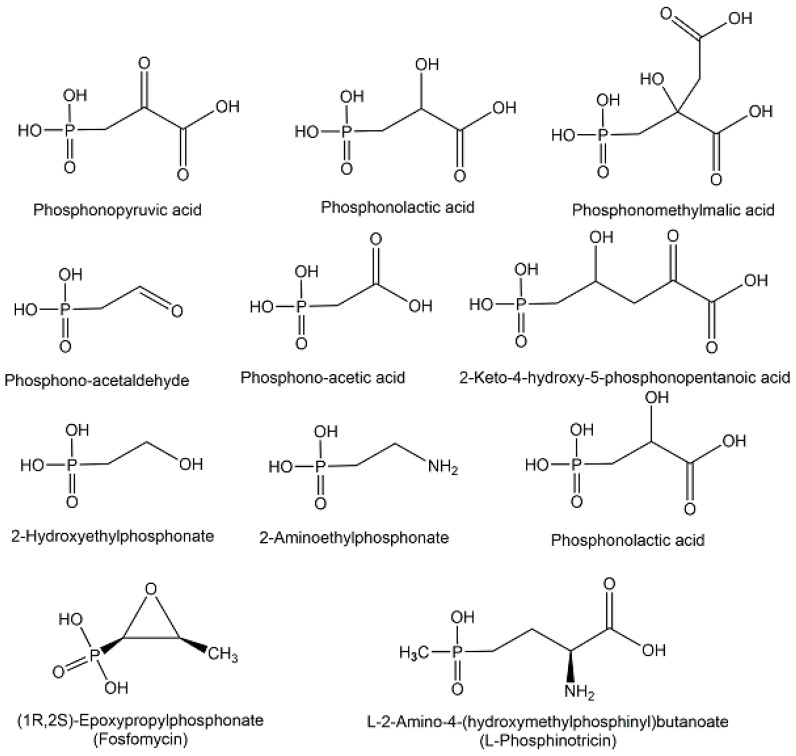
Selected microbial phosphometabolites containing phosphorus-carbon bonds.

Naturally occurring phosphonates consist of a group of microbial metabolites [40] containing one chemically and thermally stable phosphorus-carbon bond instead of a phosphorus-oxygen bond, such as phosphonolactate, phosphonomethylmalate, phos-phonoalanine, 2-aminoethylphosphonate, 2-hydroxy-ethylphosphonate, and 2-keto-4-hydroxy-5-phosphonopentanoate (see Figure 11), which originate from the key metabolites phosphonopyruvate and phosphonoacetaldehyde at early branch points of phosphonate biosynthesis [19,49]. The old broad-spectrum antibiotic (1*R*,2*S*)-epoxypropyl-phosphonate (see Figure 11), which is also named fosfomycin and still the only phosphinate antibiotic on the market, has been in clinical use for decades [50]. Fosfomycin has also been demonstrated to show antibacterial activities against bacteria which are resistant against multiple drugs and show extensive resistance [51]. The interest to fight multidrug-resistant pathogens with new antibiotics make phosphonate structures interesting candidates for selectively inhibiting pathways of pathogens by mimicking essential microbial intermediates [51].

Among the endogenous phosphinates, which contain two phosphorus-carbon bonds, the non-proteinogenic amino acid 2-amino-4-hydroxymethylphosphinylbutanoic acid, also named phosphinothricin (see Figure 11), was found in *Streptomyces* strains and has attracted much interest as an inhibitor of glutamine synthetase [40].

### 2.2. Exogenous Phosphometabolites

The role of phosphorus in non-natural compounds has attracted also much interest in drug discovery and design, medicinal, and a number of important pharmaceuticals in clinical use, such as bisphosphonates for treating bone disorders, or nucleotide analogues for treating viral diseases [52,53]. Cellular membranes are more permeable for non-charged exogenous phosphometabolites than the corresponding charged phosphometabolites, while the subsequent enzymatic formation of the biologically active charged phosphometabolites by intracellular enzymes keeps the phosphometabolites inside the cells. This is not only advantageous for the uptake of nutrients from the environment and their conversion into phosphometabolites for feeding into the metabolism of healthy cells, but also for the design of pharmaceuticals as prodrugs and their mechanism of action in the treatment of diseased cells. The phosphate moiety has a long history of FDA-approved pharmaceuticals applied in a prodrug form, from hydrocortisone phosphate approved as first prodrug in 1952 to the 17 phosphate prodrugs approved until 2022 [54]. The phosphoramidate prodrug approach, which was named as ProTide approach, was developed by the McGuigan group and uses the masking of the monophosphate or monophosphonates of nucleoside analogues for efficiently delivering into cells where, after cleavage by intracellular enzymes, the bioactive free nucleoside monophosphates and monophosphonates are released [55]. The application of this approach continues to be of interest not only for antiviral and anti-cancer nucleoside analogues [56], but also for further extensions to non-nucleoside analogues [57].

**Figure 12:**
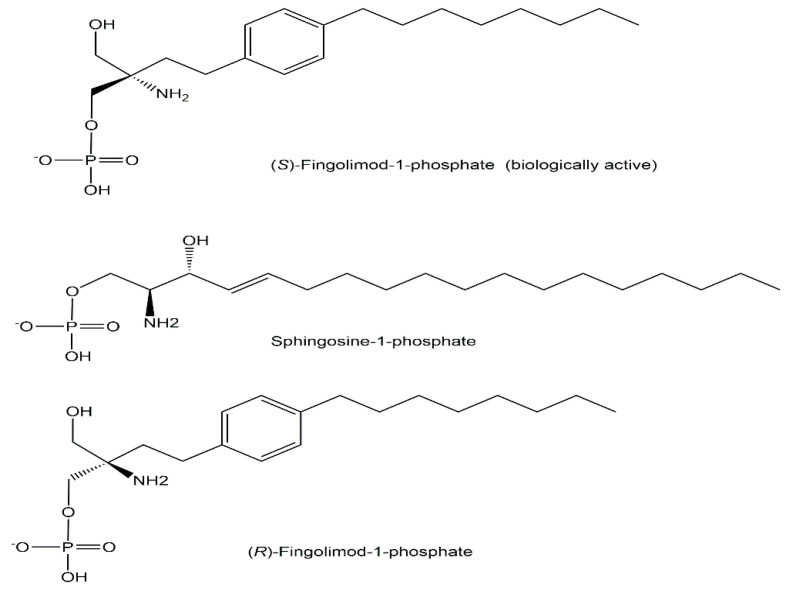
Biologically active non-natural (*S*)-fingolimod 1-phosphate, natural sphingosine 1-phosphate as well as the (*R*)-fingolimod 1-phosphate (not found in vivo).

A milestone in multiple sclerosis treatment has been reached by the discovery of fingolimod [58], first designated as FTY720, and its mechanism of action as an immunomodulatory drug acting on sphingosine-1-phosphate receptors [59,60,61]. Detailed analytical investigations demonstrated that the exogenously administrated drug is converted to the biologically active non-natural phosphometabolite (*S*)-fingolimod 1-phosphate, analogous to the sphingosine kinase-catalyzed phosphorylation of its natural counterpart sphingosine to the endogenous sphingosine-1-phosphate (see Figure 12), while the (*R*)-fingolimod 1-phosphate was not found in vivo [59,62].

Drug discovery and development based on the elucidation of the molecular effects of fingolimod as a modulator of sphingosin-1-phosphate receptors and subsequent clinical studies have led to its approval in 2010 as the first orally active pharmaceutical (brand name Gilenya) for treating multiple sclerosis relapsing forms [63]. FDA approval of Gilenya (fingolimod) in 2018 for treating relapsing multiple sclerosis in children and adolescents ages 10 years and older has represented a milestone [64].

## 3. Analysis of Biologically Active Phosphometabolites

**Figure 13:**
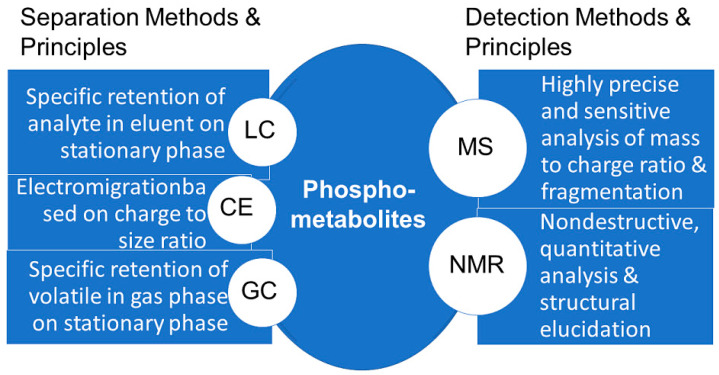
Summary of methods and principles for the analysis of phosphometabolites.

The development of suitable analytical methods for the detection of phosphometabolites has been a key prerequisite for elucidating major biochemical pathways. The investigation of photosynthetic carbon dioxide fixation required fast, general and sensitive methods for the detection of phosphate esters, which at that time were chromatography, labelling with radioactive ^14^C and ^32^P, and total phosphorus analysis [65]. The use of metabolomic technologies, systems biology, and bioactivity tools and methodologies for the identification of biologically active metabolites, for which the term activity metabolomics has been introduced [66], is also of much interest for the great diversity of biologically active metabolites which are present in cells. This great structural diversity of metabolites means that there is not just one analytical methodology for measuring all metabolites, neither in general, nor specifically for phosphometabolites, but several powerful methodologies (see Figure 13), namely high-performance separation techniques such as LC, GC, and capillary electrophoresis, and high information content detection using NMR and MS [67]. The coupling of high performance separation with high information content detection has enabled numerous advances in accurate and effective analyses of phosphometabolites, such as in the capillary electrophoresis analysis coupled to MS detection of the isomers of phosphates and pyrophosphates of *myo*-inositol [68].

### 3.1. NMR Methodologies for the Analysis of Phosphometabolites

The long and successful history of powerful NMR methodologies for analyzing and identifying molecular structures in solution is also related to the continued growth of its applications to metabolites [69]. While ^1^H NMR of common phosphorylated metabolites is straightforward, several challenges need to be overcome, such as spectra complexity and the need for standard reference databases, which have been indicated as larger chemical shift variations depending on the pH observed [70].

For nearly fifty years, ^31^P NMR has been applied for measuring phosphometabolites in cells [71] and is an obvious choice, as the properties of ^31^P and its natural abundance of 100% enable simple ^31^P reaction monitoring without stable isotope labelling procedures. The complexity of NMR spectra is significantly reduced for phosphometabolites when using ^31^P NMR in comparison to ^1^H or ^13^C NMR [72]. Dual detection of ^1^H and ^31^P NMR signals is, however, also of interest for monitoring both phosphometabolite spectra and solution pH [73]. The direct detection, validation, and determination of total concentrations from two-dimensional ^1^H−^31^P HSQC-TOCSY spectra, which is facilitated by reference data on 38 common phosphorylated-metabolites, has led to the development of an improved NMR workflow for analyzing phosphometabolites in a complex mixture [74]. Pure phosphometabolites as standard reference compounds have also been valuable in the application of ^31^P NMR for quantitatively analyzing the phosphorylated metabolites in mouse liver [75].

### 3.2. MS Methodologies for the Analysis of Phosphometabolites

The combination of powerful separation technologies with subsequent sensitive detection methodologies yielding high information content has been key for great progress in analyzing phosphometabolites. Twenty-two phosphometabolites have been analyzed reliably and selectively, with excellent linearity and sensitivity, using ion-pair reagents, such as tetrabutylammonium acetate, for HPLC separation, which was then coupled with tandem quadrupole mass spectrometry [76]. Ion-pair reagents are avoided by using ion chromatography coupled with tandem mass spectrometry, as shown by the robust quantitation of 79 phosphometabolites with a capillary ion chromatography-MS/MS method [77]. Advances in chromatographic separations, such as miniaturizing column diameters and reducing flow rates below 1 μL/min made possible the coupling to nano-electrospray ionization with improved ionization, reduced effects of ion suppression, and higher tolerance to salt concentration in the sample [78]. Tetrabutylammonium acetate has also been used as an ion-pair reagent in an LC-MS method for the fast and robust analysis of negatively charged metabolites, which has been successfully applied to more than 60 common endogenous phosphometabolites and to eight different biological extracts [79]. In order to overcome challenges in sample preparation and instrumental analysis, such as low concentrations, stability issues, ionization efficiency, recovery, and carry-over effects, chemical derivatization was introduced for analysing specific phosphorylated metabolites from the glycolysis pathway [80], twelve ribonucleotides in a single cell [81] as well as 42 phosphomonoesters of *S. cerevisiae* [82]. Improvements in phosphometabolites signal intensities, as well as the ratios of the signal to the noise, have also been achieved by column hardware optimization, such as the change from stainless steel to glass and polyethylene materials, or hybrid surface technology [83,84]. High-performance chromatography columns are essential for the separation of isomeric phosphometabolites with identical mass and charge, for example, regioisomers such as phosphatidylinositols, which are phosphorylated in the 3-, 4- or 5-position of its inositol ring [85], or enantiomers such as D- and L-glycerol-3-phosphate [86]. As matrix effects from biological samples can lead to under- or overestimation of phosphometabolites, corrections by the use of internal standards and stable isotopes have improved the reproducibility and reliability of phosphometabolite analysis in tissue extracts [87,88]. Capillary electrophoresis coupled with mass spectrometry provides a number of advantages for the analysis of the polar and charge phosphometabolites, such as high resolution, good quantitation, and low running cost [89].

### 3.3. Discovery and Structural Identification of Novel Phosphometabolites

Beyond the milestone discoveries and structural analysis of the phosphometabolites in the past of now classical metabolic pathways of biochemistry textbooks, such as glycolysis, photosynthesis, pentose phosphate, and mevalonate pathways, the further elucidation of the molecular diversity of metabolic pathways involving phosphorus used by various biological organisms in nature is a fascinating frontier for finding novel pathways and novel phosphometabolites. While preliminary analytical data may look promising for discovering novel phosphometabolites, the unambiguous structural determination of a novel phosphometabolite can be challenging.

**Figure 14:**
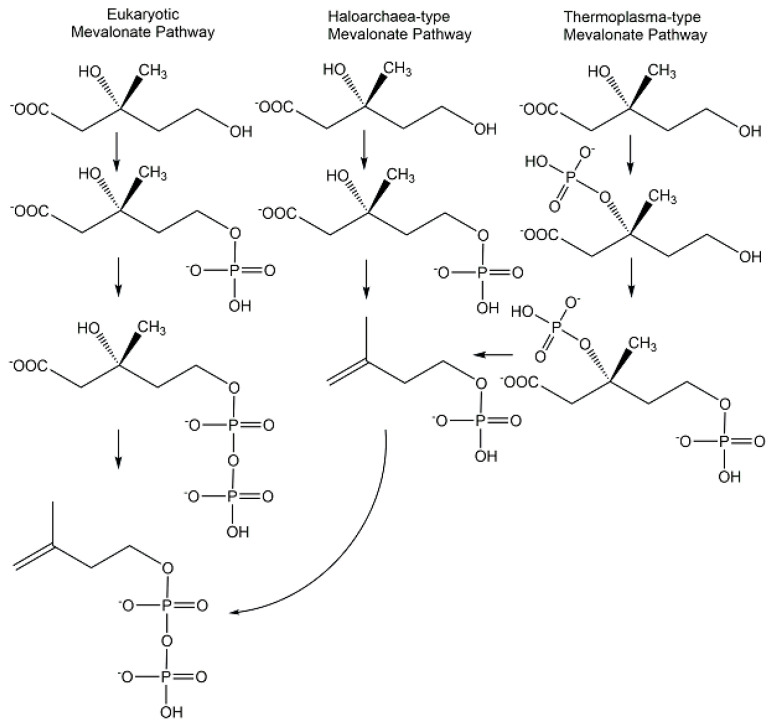
Phosphometabolites of the Eukaryotic, Haloarchaea-type, and Thermoplasma-type mevalonate pathways.

The discoveries of novel phosphometabolites in the lower mevalonate pathway have shown an unexpected molecular variety (see Figure 14) in the paths to isopentenylpyrophosphate and the importance of combining different approaches. The search for metabolites and enzymes of the classical mevalonate pathway has led to the discovery of isopentenylphosphate as an intermediate of the Haloarchaea-type mevalonate pathway [90,91]. The characterization of recombinant enzymes from *Thermoplasma acidophilum* enabled the discovery of mevalonate 3-kinase and (*R*)-mevalonate 3-phosphate instead of the classical intermediate (*R*)-mevalonate 5-phosphate and this modified mevalonate pathway is now designated as Thermoplasma-type mevalonate pathway [92].

In the recently discovered archaeal pathway of the hyperthermophilic *Aeropyrum pernix* [93,94], both the analysis and the synthesis have been essential for the structural determination of the unprecedented phosphometabolite *trans*-anhydromevalonate 5-phosphate as pathway intermediate and for the confirmation of its structural identity [95].

## 4. Synthesis of Biologically Active Phosphometabolites

After the discovery, structural identification, and analysis of biologically active phosphometabolites, their preparation as pure products of defined structure has been of key importance, as demonstrated by the prominent central phosphometabolite ATP, with the history of its discovery in 1929 and the establishment of its structure in 1935 [96], the achievement of its first chemical synthesis in 1948 [97], and various manufacturing approaches. Both non-enzymatic chemical methods, as well as biological methods using enzymes, are of continuing fundamental and practical interest, although they have been already utilized very early for the synthesis of phosphometabolites, for example, acetyl phosphate [98] and carbamoyl phosphate [99]. Whether phosphometabolites are prepared by isolation from biological resources, or chemical or biological methods of synthesis, advances in resource-efficient, selective, and straightforward methodologies continue to be of much interest [100,101]. The preparation of phosphometabolites is needed not only for confirming phosphometabolite structures but also for providing standards, analogues, or stable-isotope-labelled phosphometabolites, and for further investigations of their biological activities.

### 4.1. Chemical Methods of Synthesis

**Figure 15:**
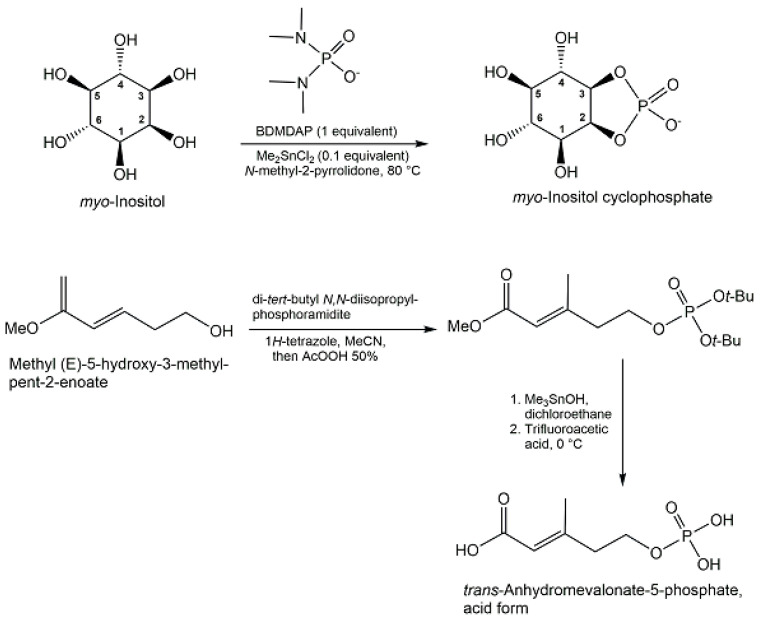
Chemical syntheses of *myo*-Inositol cyclophosphate, and *trans*-Anhydromevalonate-5-phosphate.

A robust phosphorylation strategy in aqueous solution has been demonstrated with diamidophosphate, which has not only been useful for efficient regioselective α-phospho-rylation of glycolaldehyde and D-glyceraldehyde, but also for the synthesis of aldose 1,2-cyclic phosphates, such as D-erythrose 1,2-cyclophosphate from D-erythrose in 87% yield, and D-threose 1,2-cyclic phosphate from D-threose in 80% yield [102]. The synthesis of *myo*-inositol cyclophosphate has been achieved in 80% yield (see Figure 15) from myo-inositol by regioselective one-pot cyclophosphorylation using bis-(dimethylamino)phosphorodiamidate [103]. Process analytical technology has been shown to provide advantages for synthesizing acetyl phosphate lithium salt in high purity [104].

**Figure 16:**
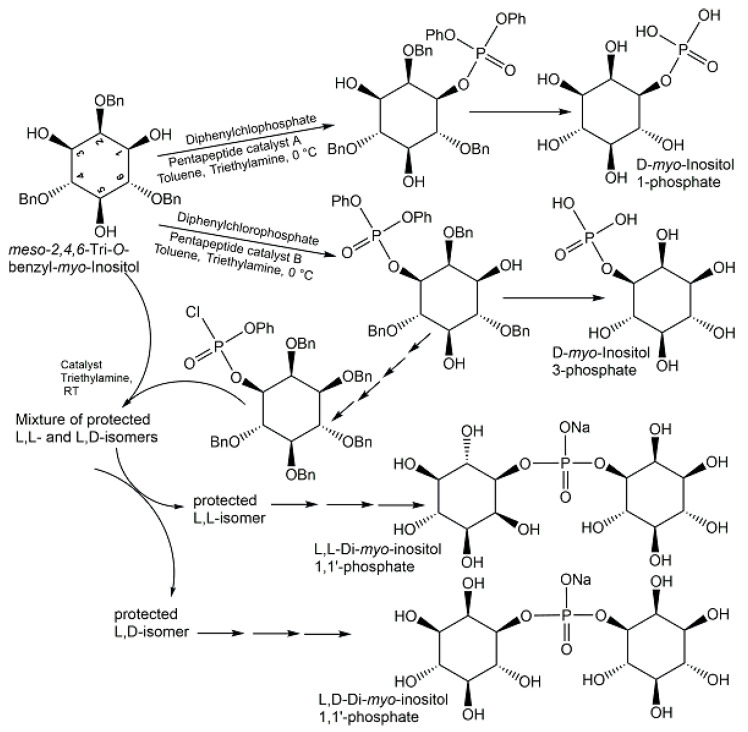
Chemical synthesis of D-*myo*-inositol 1-phosphate, D-*myo*-inositol 3-phosphate, L,L-, and L,D-di-*myo*-inositol 1,1′-phosphate.

Starting from methyl tetrolate, the first chemical synthesis of *trans*-anhydromevalonate 5-phosphate (see Figure 15) has been achieved in six reaction steps [95]. In the first three reaction steps, methyl tetrolate was first converted to methyl (*E*)-5-hydroxy-3-methyl-pent-2-enoate, which was then reacted with di-tert-butyl *N,N*-diisopropyl-phosphoramidite, THF, and 1H-tetrazole to form the phosphite intermediate, which was subsequently oxidized with peracetic acid [95]. The removal of the methyl group of the ester was done with trimethyltin hydroxide in 1,2-dichloroethane, followed by chromatographic purification of the protected product, and then *trans*-anhydromevalonate 5-phosphate was obtained in the acid form (see Figure 15) after the tert-butyl protecting groups were removed with trifluoroacetic at 0 °C in methylenchloride as solvent [95]. Alkenes and phosphoric acid diesters as the phosphate source have been the starting materials for the direct synthesis of allylic phosphate esters in good yields, using a newly developed aerobic phosphatation-using dichloroethane as a solvent and catalyzed by a dual catalyst system consisting of 10 mol % diphenyldiselenide as selenium π-acid catalyst and 10 mol % 2,4,6-Tris(4-methoxyphenyl)pyryliumtetrafluoroborate as a photocatalyst, with irradiation at 465 nm [105]. Histidine-based peptide catalysts have been successfully utilized for the synthesis of various phosphometabolites (see Figure 16), such as D-*myo*-inositol 1-phosphate and D-*myo*-inositol 3-phosphate with >98% ee [106], the chiral L,L-form, and the meso L,D-form of di-*myo*-inositol 1,1′-phosphate [107].

The synthesis of three teicoplanin A_2_-2 analogs has been achieved by selective catalytic phosphorylation of the 6-hydroxy group of the N-decanoylglucosamine, the mannose, or the *N*-acetylglucosamine moiety of its parent glycopeptide, using three specifically developed peptide-based catalysts, excess diphenylphosphorylchloride as phosphorylating agent, 1,2,2,6,6-pentamethylpiperidine as a base and tetrahydrofuran/methylenchloride as a solvent [108]. Diastereomeric and cyclic dinucleotides have been prepared by stereo-controlled synthesis in organic solvents using 20 mol % of chiral phosphoric acid catalysts which are either derived from peptides and phosphothreonine, or from a 1,1′-binaphthyl-2,2′-diol enantiomer [109].

### 4.2. Biological Methods of Synthesis

The molecular logic of the biotransformations by which biological cells prepare, just in time and where needed, key phosphometabolites acting as cofactors and cosubstrates is of great interest for cell metabolism [13] and for designing novel methods for their synthesis.

ATP synthase-catalyzed ATP formation is the biocatalytic molecular machine providing significant amounts of ATP which are synthesized and needed daily by biological organisms [110,111]. Although the exploration of various artificial stimuli-responsive biocatalytic ATP synthesis systems is attractive [112] (for example, proteoliposome systems containing oriented bacteriorhodopsin and using light for proton gradient generation to drive the formation of ATP [113]), further improvements of rate and stability of ATP production achieved by these approaches are needed. Therefore, industrial large- scale production utilizes *Corynebacterium ammoniagenes* whole cells for the biotransformation of adenine and inorganic phosphate to ATP [114]. Continuous ATP production also has many interconnections with biocatalytic synthesis of phosphometabolites and various biocatalytic systems using high-energy phosphoryl donors and suitable phosphotransferases have been developed, starting from ADP, AMP, or adenosine [115].

**Figure 17:**
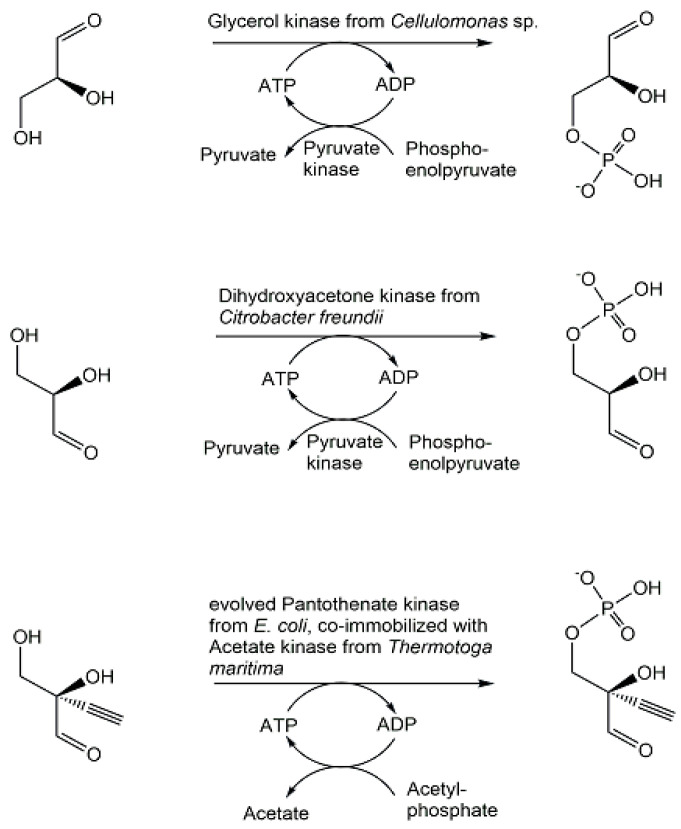
Enzymatic synthesis of phosphorylated chiral glyceraldehydes.

As key phosphometabolites of central biochemical pathways serve essential needs as enzyme substrates, cosubstrates, cofactors, or standards, their synthesis, and purification are highly important. Selective methods for synthesizing not only racemates but also the pure enantiomers of chiral phosphometabolites are essential for investigating their properties, functional roles, and the influence of chirality. In addition, the biocatalytic methods of synthesis and their natural scope are of much interest for exploring and widening the capabilities of metabolic enzymes. (2*S*)-glyceraldehyde 3-phosphate has been synthesized by biocatalytic phosphorylation of (2*S*)-glyceraldehyde (see Figure 17) using glycerol kinase [116,117], while preparing (2*R*)-glyceraldehyde 3-phosphate required the enantiocomplementary dihydroxyacetone kinase for catalyzing the phosphorylation of (2*R*)-glyceraldehyde [118]. The synthesis of an unnatural analogue substituted with a triple bond at the C2-position, 2-ethynyl-(2*R*)-glyceraldehyde 3-phosphate, a key islatravir intermediate, was achieved by phosphorylating 2-ethynyl-(2*R*)-glyceraldehyde (see Figure 17), with complete conversion at 0.2 M concentration, using a kinase which after the initial discovery of a low activity pantothenate kinase from *E. coli*, directed evolution and further engineering of the enzyme was obtained as a kinase variant with 100-fold better activity [119].

**Figure 18:**
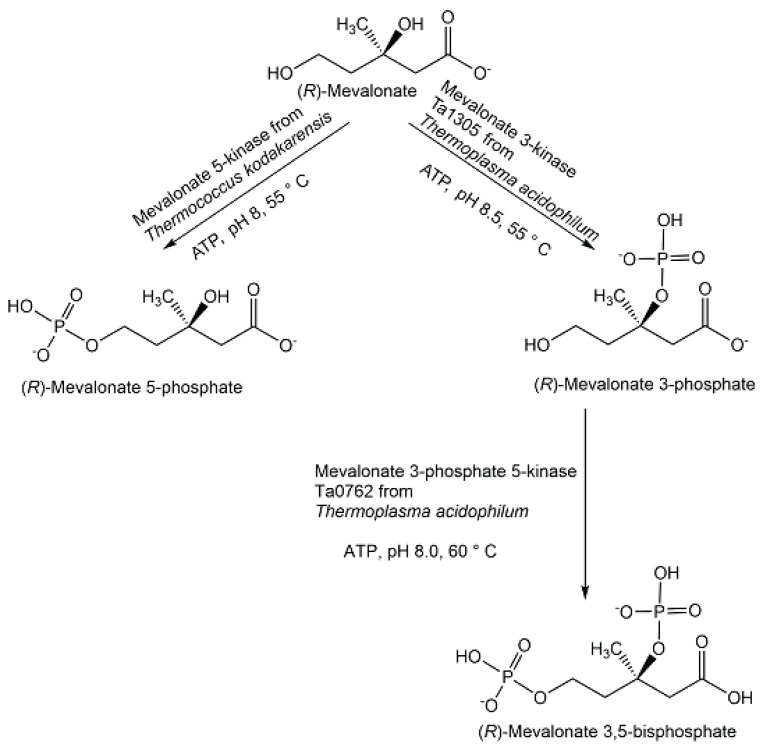
Enzymatic synthesis of phosphorylated chiral mevalonates.

Chiral phosphohydroxycarboxylic acids are important phosphometabolites in glycolytic, mevalonate, and shikimate pathways. The glycolysis intermediate D-glycerate 2-phosphate was prepared with high purity and good yield by glycerate 2-kinase-catalyzed phosphorylation of D-glycerate [120]. The classical mevalonate pathway intermediate (*R*)-mevalonate 5-phosphate (see Figure 14) has been also been synthesized with high purity and good yield by mevalonate 5-kinase-catalyzed phosphorylation (see Figure 18) of either the (*R*)-enantiomer or the racemic form of mevalonolactone [121]. The novel phosphometabolites (*R*)-mevalonate 3-phosphate and (*R*)-mevalonate 3,5-bisphosphate from the recently discovered Thermoplasma-type mevalonate pathway (see Figure 14) were prepared at a very small scale by the use of novel kinases (see Figure 18) and have been found to be stable.

(*R*)-mevalonate 3-phosphate was synthesized from (*R*)-mevalonate by phosphorylation catalyzed by mevalonate 3-kinase Ta1305 [94,122]. (*R*)-mevalonate 3,5-bisphosphate has been prepared from (*R*)-mevalonate by a first phosphorylation catalyzed by mevalonate 3-kinase Ta1305, followed by a second phosphorylation catalyzed by mevalonate 3-phosphate 5-kinase Ta0762 [123,124], or by the E140 mutants which convert the mevalonate 3-kinase into a mevalonate 3-phosphate 5-kinase by replacing a glutamate residue interacting with the substrate by smaller amino acids [124]. Shikimate-3-phosphate lithium salt was prepared with >97% purity and 53% yield by highly efficient and selective phosphorylation of shikimate in one reaction step catalyzed by recombinant shikimate kinase AroL from *E. coli* [125,126]. Phosphorylated monosaccharide sugar acids represent an important family of biologically active phosphometabolites which play key roles in carbohydrate metabolism and illustrate also the higher molecular diversity of chiral phosphohydroxycarboxylic acids as the number of carbon atoms increases. Various approaches have been taken for the synthesis of phosphorylated monosaccharide sugar acids, such as the use of microbial whole cells, selective enzymatic phosphorylation of the corresponding monosaccharide sugar acid, or selective water elimination from phosphorylated monosaccharide sugar acids. This is exemplified with 2-keto-3-deoxy-6-phospho-D-gluconate, abbreviated KDPG, which was prepared from *Alcaligenes eutrophus* strain H16 F34 lacking KDPG-aldolase activity [127], from 2-keto-3-deoxy-D-gluconate by kinase-catalyzed phosphorylation [128,129], and through 6-phosphogluconate dehydratase-catalyzed water elimination from 6-phospho-D-gluconate [130].

Enantiomerically pure D-xylulose 5-phosphate and L-xylulose 5-phosphate, which have been synthesized by 5-phosphorylation of the corresponding xylulose catalyzed by recombinant D- and L-xylulokinases [131,132], are occurring in different metabolic pathways, as well as both together in the same pathway of pentose and glucuronate interconversions, where they are separated by the two reactions of the epimerization in the 3- and the 4-position. Eight phosphorylated ketopentoses have been synthesized in 84–96% yield at gram scale using a multi-enzyme cascade reaction involving isomerization or epimerization coupled with phosphorylation catalyzed by specific monosaccharide kinases, whereby ATP was not recycled and silver nitrate precipitation was used for the removal of the byproducts [133]. D-tagatose 1,6-diphosphate, an important phosphometabolite at the intersection of different metabolic pathways of monosaccharides and in tagatose pathways in pathogenic bacteria, has been prepared as lithium salt at gram scale by efficient and scalable LacC-catalyzed phosphorylation of D-tagatose 6-phosphate [134,135]. Multi-enzymatic reaction cascades were utilized for synthesizing terminally phosphorylated D-monosaccharides [136] and four terminally phosphorylated L-monosaccharides [137]. A promising method for preparing many phosphate-containing cofactors and their intermediates is the utilization of suitable metabolic enzymes, for example, the transaminase-catalyzed synthesis pyridoxamine-5′-phosphate from pyridoxal-5′-phosphate [138], or the nicotinamide riboside kinase-catalyzed phosphorylation of nicotinamide riboside to nicotinamide mononucleotide [139].

**Figure 19:**
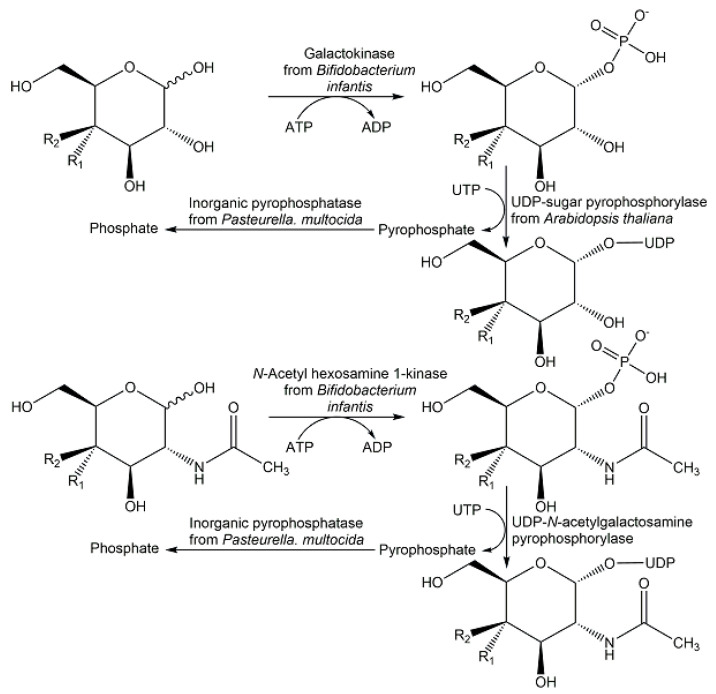
Enzymatic synthesis of uridine 5′-diphosphate sugars as examples of nucleotide sugar synthesis by using enzymes of the salvage pathway.

Scalable multi-step enzyme processes towards nucleotide sugars via de novo and salvage pathways have attracted much interest, whereby the trend to utilize the salvage pathway enzyme has increased [140]. Twelve nucleotide sugars have been synthesized at gram scale from monosaccharides at a 0.2 M starting concentration by the two enzymatic reaction steps of phosphorylation and pyrophosphorylation (see Figure 19) in a multi-enzyme system with 52–97% yield [141].

*N*_ω_-phospho-L-arginine, an important energy reserve metabolite providing fast energy supply for critical needs of invertebrates and parasites, has been efficiently synthesized by selective ArgK-catalyzed phosphorylation of L-arginine using phosphoenolpyruvate and pyruvate kinase for regenerating ATP [142,143].

**Figure 20:**
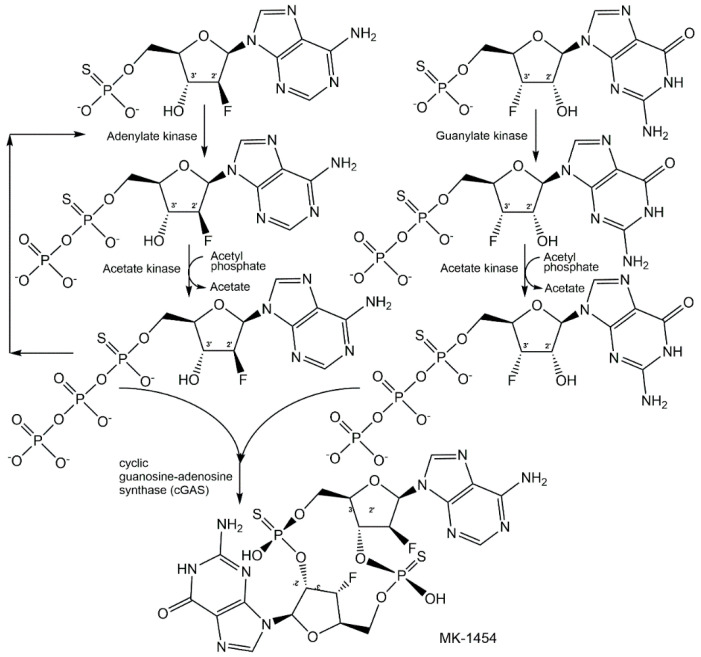
Biocatalytic synthesis of a single diastereomer of the non-natural cyclic dinucleotide MK-1454 with the desired stereochemistry of the *R*_p_/*R*_p_ phosphorothioate linkage.

As cyclic dinucleotides are phosphometabolites with important biological activities, simple, safe, and scalable biotechnological methods for their synthesis have attracted much interest [144,145], because the chemical methods of synthesis are non-sustainable and time-consuming. The attractiveness of analogues of cyclic dinucleotides with improved therapeutic properties can add even further aspects for embarking on biocatalytic methods of synthesis, as very nicely demonstrated by a joint Merck-Codexis team for analogues of cyclic guanosine monophosphate-adenosine monophosphate (cGAMP) [146,147,148]. As cyclic dinucleotide analogues as phosphorothioates showed improved stability and increased cellular uptake, it was essential to investigate the phosphorus stereochemistry in early discovery efforts, and among the four diastereomers of dithio-cGAMP the *R*_p_/*R*_p_-diastereomer MK-1454 has been found to have the highest bioactivity [147]. The process for synthesizing MK-1454 using a cascade reaction in one pot (see Figure 20) started with the enzymatic *P*-desymmetrization of 2′-fluoro-thioAMP and 3′-fluoro-thioGMP, using adenylate kinase and acetate kinase for generating 2′-fluoro-(*S*_p_)-thioATP, and guanylate kinase and acetate kinase for realizing 2′-fluoro-(*S*_p_)-thioGTP, with acetyl phosphate lithium salt as phosphoryl donor, until the kinase reaction at pH 7.4 and 10 °C was completed after 17 h [147]. The conditions in the reactor were then changed to optimal conditions for the subsequent cyclization reaction, which was catalyzed by cyclic guanosine-adenosine synthase (cGAS) at pH 7.8 and 35 °C for 24 h, and the single diastereomer MK-1454 was isolated with 62% yield [147]. The excellent selectivities of the final engineered enzymes, the tools and methodologies for directed evolution and engineering of enzymes, and reaction engineering have enabled a resource-efficient process without the isolation of intermediates.

## 5. Outlook

The analysis of biologically active metabolites has come a long way in extending the frontiers of knowledge on stable and reactive phosphorus compounds, condensed phosphates, endogenous, and exogenous phosphometabolites. Inorganic and organic phosphorus-containing small molecular weight compounds continue also to be of much interest as bridging interfaces between the living and the non-living, the biotic and the prebiotic world. In addition to the discovery of phosphometabolites and the identification of their correct structures, the analysis of their transformations can be very valuable for elucidating more molecular details of the biogeochemical flows of phosphorus, one of the planetary boundaries identified to be concerned about at global and regional levels [2]. The identification of chemical and biological reactions involving phosphometabolites can also provide inspiration for designing and developing novel in vitro synthetic methodologies. The correct assignment of gene sequences coding for natural enzymes which catalyze reactions involving phosphometabolites, as well as the development of novel engineered and evolved enzymes catalyzing reactions of phosphometabolites, can also be key enablers of novel synthetic approaches. The synthesis of phosphometabolites has progressed very well and numerous phosphometabolites have become accessible in pure form for the first time. Nevertheless, there is much more work ahead for developing scalable syntheses of known and novel phosphometabolites, reducing the complexity of synthetic routes [149], improving the molecular economy, and enabling sustainable phosphorus chemistry [150].

## Data Availability

Not applicable.
